# A Trusted Lightweight Communication Strategy for Flying Named Data Networking

**DOI:** 10.3390/s18082683

**Published:** 2018-08-15

**Authors:** Ezedin Barka, Chaker Abdelaziz Kerrache, Rasheed Hussain, Nasreddine Lagraa, Abderrahmane Lakas, Safdar Hussain Bouk

**Affiliations:** 1 College of Information Technology, United Arab Emirates University, P.O. Box 17551, Al Ain 15551, UAE; ebarka@uaeu.ac.ae (E.B.); alakas@uaeu.ac.ae (A.L.); 2 Department of Mathematics and Computer Science, University of Ghardaia, Ghardaia 47000, Algeria; 3 Laboratoire d’Informatique et de Mathématiques, University of Laghouat, BP 37G, Route de Ghardaia, Laghouat 03000, Algeria; n.lagraa@lagh-univ.dz; 4 Institute of Information Systems, Innopolis University, Innopolis 420500, Russia; r.hussain@innopolis.ru; 5 Department of Information and Communication Engineering, Daegu Gyeongbuk Institute of Science and Technology (DGIST), Daegu 42988, Korea; bouk@dgist.ac.kr

**Keywords:** UAV, FANET, NDN, VANET, trust, energy efficiency

## Abstract

Flying Ad hoc Network (FANET) is a new resource-constrained breed and instantiation of Mobile Ad hoc Network (MANET) employing Unmanned Aerial Vehicles (UAVs) as communicating nodes. These latter follow a predefined path called ’mission’ to provide a wide range of applications/services. Without loss of generality, the services and applications offered by the FANET are based on data/content delivery in various forms such as, but not limited to, pictures, video, status, warnings, and so on. Therefore, a content-centric communication mechanism such as Information Centric Networking (ICN) is essential for FANET. ICN addresses the problems of classical TCP/IP-based Internet. To this end, Content-centric networking (CCN), and Named Data Networking (NDN) are two of the most famous and widely-adapted implementations of ICN due to their intrinsic security mechanism and Interest/Data-based communication. To ensure data security, a signature on the contents is appended to each response/data packet in transit. However, trusted communication is of paramount importance and currently lacks in NDN-driven communication. To fill the gaps, in this paper, we propose a novel trust-aware Monitor-based communication architecture for Flying Named Data Networking (FNDN). We first select the monitors based on their trust and stability, which then become responsible for the interest packets dissemination to avoid broadcast storm problem. Once the interest reaches data producer, the data comes back to the requester through the shortest and most trusted path (which is also the same path through which the interest packet arrived at the producer). Simultaneously, the intermediate UAVs choose whether to check the data authenticity or not, following their subjective belief on its producer’s behavior and thus-forth reducing the computation complexity and delay. Simulation results show that our proposal can sustain the vanilla NDN security levels exceeding the 80% dishonesty detection ratio while reducing the generated end-to-end delay to less than 1 s in the worst case and reducing the average consumed energy by more than two times.

## 1. Introduction

Flying ad-hoc network (FANET) employing Unmanned Aerial Vehicles (UAVs) as nodes is a new breed of Self-Organized Networks (SONs) offering a plethora of innovative and exciting applications including, but not limited to, Disaster Management [1] and public safety insurance [2]. Without loss of generality, like other breeds of SONs, FANET applications are also based on cooperation-aware communication where information is exchanged among communicating nodes. This phenomenon advocates for content-centric communication paradigm for FANET applications where the information is of the essence than the source and/or location of information. As a result, owing to the Future Internet Architecture (FIA), recently content-centric networking (CCN) and named data networking (NDN) have been considered to be the architectural backbones for SONs and its breeds, such as VANET-based and FANET-based clouds [3,4] and flying social networks [5]; however, this paper only focuses on FANET.

Among other challenges for most of SONs and mainly FANETs, security and privacy are two of the most daunting challenges due to several reasons such as resource-constraints, mobility, stringent delay requirements, and so forth. These characteristics make FANET more susceptible to security attacks. To date, security and privacy issues related to FANET have not been given ample attention in the literature and need thorough investigation. Owing to the nature of FANET applications, security of the content and its communication through the network are of paramount importance and prone to content-related attacks. To this end, several solutions have been proposed to secure the content communication and guarantee trusted delivery of the exchanged messages [6,7]. Nevertheless, finding a balanced trade-off among security, efficiency, and the network requirements is still an open challenge. Security solutions for SONs communication including FANET can be divided into two categories: trust-based solutions [8] and cryptography-based solutions [9].

Traditionally, FANET applications require content/data exchange among the network entities where the location of content producer(s) and/or provider(s) is of least importance. Therefore, NDN can be the key enabler for the realization of FANET application that not only enriches the applications space but also aids in solving the traditional problems faced by traditional TCP/IP-based networks such as mobility management, addressing, reliability, and security [10]. However, NDN suffers from new security issues such as interest flooding, cache poisoning, access control, and data authenticity, to name a few [11]. Existing solutions in the literature consider traditional security approaches adopted in the vanilla NDN for fixed or wired networks [12,13]. In the current solutions, the content is signed by the producer and then by the authority that issued the certificate to the producer. The certificate signing is continued in the chain of trust until it reaches a self-signed network entity (conventionally trusted authority). This mechanism of verifying and ensuring the data authenticity aids in improving the data trustworthiness. However, in a highly dynamic and energy-restricted FANET environment, it incurs a huge overhead which is unacceptable for the delay-sensitive and bandwidth and energy consuming applications [14]. To this end, a trusted lightweight communication architecture respecting both the NDN security principles and FANET features is essential. Therefore, the goal of this paper is to propose an efficient and lightweight trust-aided communication mechanism for FNDN where trust evaluation aids the data authenticity. To fill the voids, we propose a new Monitor-based communication architecture for FNDN that reduces the authentication processing delays and bandwidth consumption for the inter-UAV communications. The proposed mechanism maintains the similar vanilla NDN authentication process for the data forwarded through the designated Ground Stations. The proposed architecture uses our FANET monitoring solution [15] which selects the most trusted and stable UAVs as network monitors. These monitors are responsible for interest packets dissemination, gathering, and forwarding to the next monitor. When a node requires content(s) and generates interest for it, the interest eventually reaches the node that has the content in its CS (either it is the owner of the content or has received the content during past communication). The content will be pushed back to the requester (through the reverse path) from which the interest packet has been received.

To achieve the goal of trust-based communication, first inter-UAV trust is established through evaluation of the historical communications among UAVs based on actions similarities. Then, instead of authenticating the chain of trust or web of trust (which is both time consuming and inefficient), we minimize the overhead by evaluating the data producer’s trust. Trust value is managed for every producer and the producers are classified based on their level of trust. For the trusted producers, we perform probabilistic authenticity verification where authenticity is checked and verified for a fraction of the content generated by that producer whereas for non-trusted producers, the authenticity of every chunk of the content is verified. It is worth noting that with the increase in trust value of the producer, the number of verifications will decrease thereby increasing performance. Finally, for the data coming from the vanilla NDN in fixed or wired networks, the authentication process is automatically embedded into the intermediate routers until the content reaches the fixed Ground Station that are considered as trusted middle-ware to the trusted authorities, therefore there is no need to check it again. As a result, it minimizes the authentication delay and supports the FANET’s real-time applications. The main contributions of this paper are summarized as follows:

- A new trust-based communication architecture is proposed for FNDN.
- A lightweight data authentication strategy is used over the established inter-UAV trust.
- A monitor-based interest forwarding and data delivery technique through the shortest and most trust path is proposed.

The remainder of the paper is organized as follows: in Section 2, we present the existing trust establishment works for FANET, and the data authentication process used by the vanilla NDN. Section 3 describes our Trust-establishment and Monitor selection strategy, followed by the proposed trust-based lightweight authentication solution Section 4. The performance evaluation of our proposal is discussed in Section 5 followed by open security challenges in FNDN in Section 6. Section 7 concludes the paper with future directions.

## 2. Background and Related Works

Trust establishment in mobile networks is essential for the realization of efficient secure applications. To date, various solutions have employed trust modeling mechanisms to enhance the inter-vehicle communications for MANET, VANETs, and FANET. In this section, we present the existing approaches for trust in FANET, as well as an overview of Vanilla NDN components.

### 2.1. Trust Management in MANET and VANET over NDN

Trust establishment and management are well studied in distributed environments, such as MANETs and VANETs [16,17]. Trust models are generally classified into entity-based, data-based, and hybrid models depending on the revocation target. In addition, there also exist trust establishment solutions for both MANET and VANET contexts.

Lu et al. [18] propose a social trust-based security scheme to verify the public-key and producer identity binding in Mobile Information Centric environment. Their proposal allows user to verify the content producer identity and its public-key binding relationship by retrieving the identity bundle from a trust social network. However, as revealed by the authors, this proposal works only for low mobility scenarios. In addition to the trust establishment issues, Talebifard et al. [19] presented the features of information-centric vehicular networks together with the main open challenges. Ahmad and Adnane [20] proposed the concept of Context Aware Routing Protocol (CARP) in NDN based VANETs which aims to transmit trusted information in a secure and reliable way. This proposal is designed such that it supports the traditional NDN Daemon in every vehicle, consisting of NDN data structures and forwarding strategies, and implements rules to check the trustworthiness of messages. However, this proposal sustains the same NDN hierarchical data authentication process which is not suitable for highly dynamic environment like VANET. Through extensive simulations, Jain et al. [21] conclude that the classical geo-based routing protocols do not sustain the same performance in vehicular named data networks as in VANETs, that is why they propose to use a trust-based routing strategy instead. However, they only discussed the expected performance of their solution without implementing the proposal.

### 2.2. Trust Management for FANET

As mentioned in the previous section, the concepts of trust management, interaction-based trust, and recommendation-based trust have been largely studied in the literature for different mobile environments. However, only few solution have been proposed for FANET environment. Furthermore, to the best of our knowledge, none of the previous work have studied the trust establishment for FANET over Named Data Networking.

Moahmmed et al. [22] analyzed the requirements for efficient UAV communication. They identified the similarities and differences between MANETs and FANETs, and discussed various trust-based protocols and management schemes that can be used in FANET. However, this work should be improved to consider different mobility patterns, propagation models, and energy restrictions before its implementation. Furthermore, Yuan et al. [23] presented a trust-based connectivity analysis between to FANETs’ nodes. Although, these two networks nodes are within the range of each other, a link is considered existent only if the estimated trust value is higher than a predefined threshold. Numerical results show that the proposed scheme is effective in ensuring a reliable data delivery path. The main limitation of this scheme is that it works only in high UAVs density cases and requires an important trust establishment delay due to the required learning phase.

In our previous work [24], we proposed a trust management architecture distinguishing the intentional and unintentional misbehavior in FANETs. The main idea is to evaluate for all neighboring UAVs: the buffer occupancy, remaining energy, and mobility patterns which are the main reasons of the unintentional misbehavior. Thus, it enables to make a more accurate decision regarding the UAVs behavior. However, this proposal works only for the IP-based classical networks and not for Information Centric Networks (ICNs).

In [25], the authors proposed a fuzzy classification trust model (FCTM) for FANETs. The proposed scheme is based on node’s behavior and collaboration in the network operations. They also used Quality of Service (QoS) and social parameters to enhance the trust evaluation of each UAV. However, same as the previous work, this work is an entity-centric approach and do not work for ICNs.

### 2.3. Components of Vanilla NDN

Communication in vanilla NDN involves two types of packets (i) Interest packet that requests the content from the network and contains the name of the requested content. Interest packet consists of various fields such as Name, Nonce, Selectors, InterestLifeTime, and ForwardingHint. Name and Nonce are mandatory fields in an interest packet, whereas the rest of the fields are optional. Selectors are used to find the best match among the available data with the requested data in the interest. Finally, the lifetime and the forwarding hint are additional optional information provided by the requester. (ii) Data packet is a reply to the interest packet containing the actual requested content. Unlike the interest packet that explores the network looking for the desired content using a given forwarding strategy, the data packet is returned in a unicast manner and the data owner has to sign every single packet to prove the authenticity of the data [26].

Every node in the vanilla NDN (a specific implementation of NDN) maintains the following three data structures:

- Content Store (CS): Every node maintains a local cache memory to store the received content from other nodes. This node may be the requester or the intermediate relayer along the path. With CS, the local availability of content guarantees increased performance and reduces the delay.
- Pending Interest Table (PIT): PIT stores all the interests that a router has forwarded but not yet satisfied.
- Forwarding Information Base (FIB): It is a routing table matching the requested data name with its corresponding interface.

**Figure 1:**
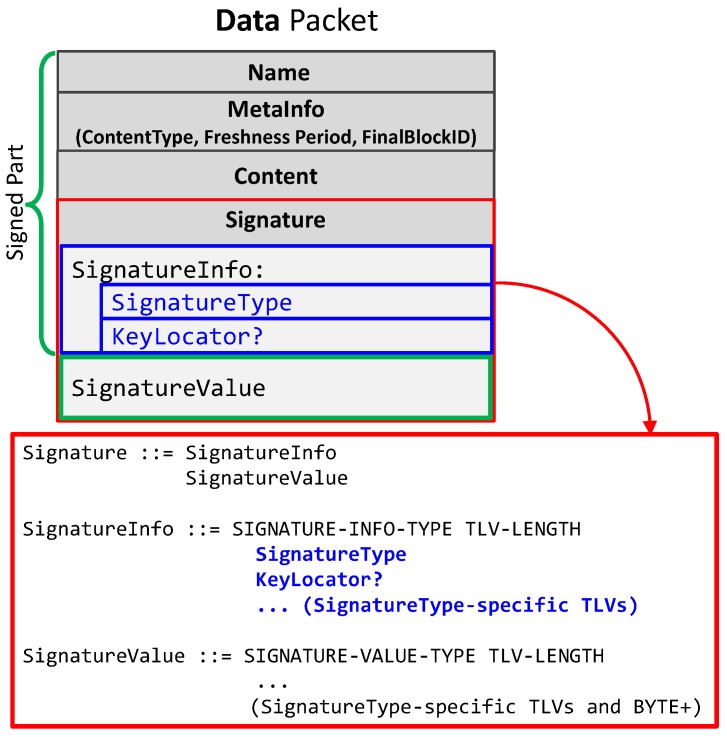
Format of NDN data packets [27].

NDN provides intrinsic security where the data itself is secured rather than the communication channel (as in the traditional networks) [8]. Each content is signed by the respective producer(s). Intrinsic security on one hand avoids the resource consuming complex mechanisms required for communication security and on the other hand makes the authenticated data locally available in the CS. Hence, most of the data-related attacks are automatically mitigated. The signature of a given data packet has two main fields, i.e., the signature type (generally SHA-256 with RSA), and the KeyLocator representing the name of the producer’s public key. It specifies the name that points to another data packet containing the certificate or the public key needed to validate the signature value. Since the certificate is itself an NDN data packet, it has a signature field of its own. Figure 1 shows the format of an NDN data packet.

Data packet in NDN is hierarchically checked for authentication. For instance, if a node receives a FANET course produced by an author ’A’ and published in a blog ’LIM’, the authentication of this packet is carried out using the following steps:

1. Check the signature of the author,
2. Check the signature of the blog administrator who certified the author, and
3. Check the authority who certified the blog administrator. This latter is generally a trusted self signed authority, such us Google or Yahoo.

**Figure 2:**
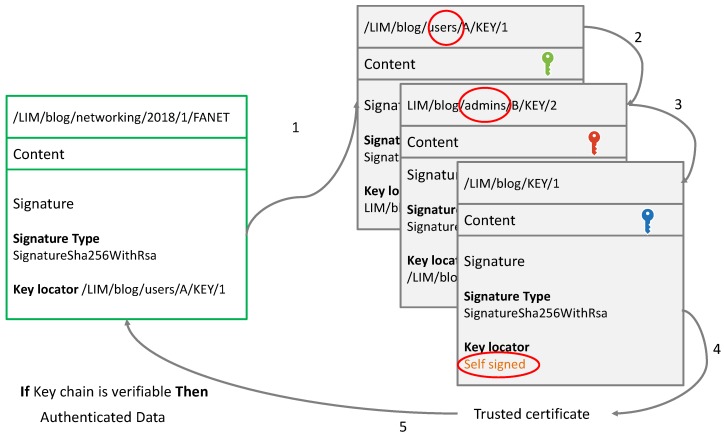
Vanilla NDN hierarchical data authentication process [27].

If this whole key chain is verifiable, then the data packet is considered authentic, as shown in Figure 2.

## 3. Trust Establishment and Trust-Based Monitor Selection

The main idea of this work is to establish trust among the communicating UAVs and select the most trusted UAV (called monitor) with high probability of data holding and having enough energy for the rest of its mission. Another goal is to take advantage of the selected monitor to avoid the broadcast storm problem that may be caused by the dissemination of interest packets. When the data producer or a node having a copy of the requested data is found, it sends back the data packet to the requester UAV through the shortest and most trusted path as used in [28]. Finally, the data authentication process is carried out based on how trustful is the producer of this data.

In the following sections, we first present the proposed inter-UAV trust establishment and management, and then outline a lightweight data authentication based on inter-UAV trust.

### 3.1. Trust Establishment and Link Duration Estimation

**Figure 3:**
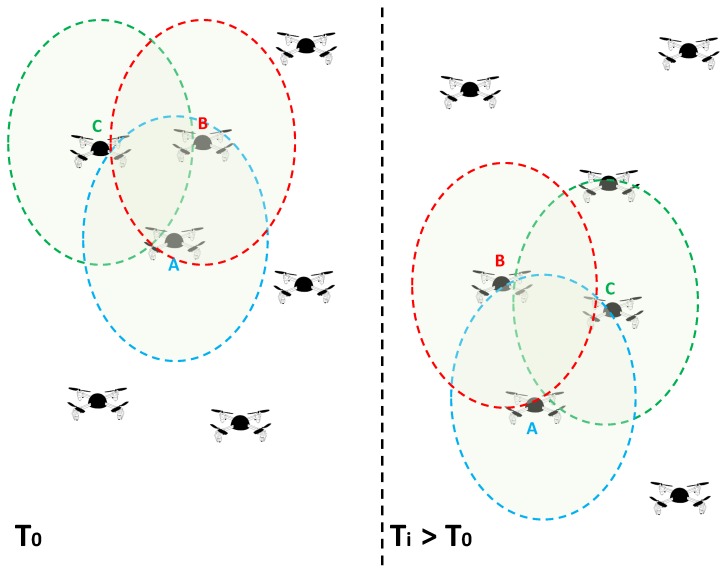
UAVs trusting each other and moving with similar mobility patterns.

A UAV *A* is considered friend of another UAVs *B* and *C* if the UAV *A* has a high trust value from the perspective of *B* and *C*, and the communication medium is mostly stable between them, as illustrated in Figure 3. Therefore, node *A* is included in the friends table only if its Friend Score (FS) is higher than a predefined threshold; this node will also be removed from the table once its FS(i,j) value goes below the same predefined threshold. To compute FS, we use the following equation:

(1) FS(i,j)=Trust(i,j)×LD(i,j)

A node is considered as a friend when its FS is greater than a certain threshold $FS_{th}$. In addition, Trust(i,j) and LD(i,j) represent the trust evaluation of a UAV *i* about another UAV *j*, and the link duration between these two nodes, respectively. Details about the computation of both metrics are provided in the following sections.

#### 3.1.1. Inter-UAV Trust Establishment

When focusing on inter-UAV trust, we generally distinguish between two metrics: direct trust and indirect trust. Direct trust can be defined as the local knowledge-based evaluation of the direct interactions among UAVs, whereas indirect trust is the evaluation of the direct interactions between two UAVs based on the opinions of other UAVs about the honesty of the two participant UAVs (also referred to as recommendation-based evaluation). Since direct trust is more relevant than indirect trust when the number of interactions (#int) increases, our UAV-to-UAV trust levels are adapted using the following factor: $\frac{1}{\#int\;+\;1}$; this way, if we have more interactions, we assign a higher weight to direct trust than to indirect trust, and vice versa. In addition, UAVs may also rely on Ground Stations (GSs) if they are available in their communication range. In this case, the opinion of the GS is directly considered instead of the UAV local evaluation. In FANETs, GSs are generally represented by the (Air Traffic Controllers). Equation (2) shows how the inter-UAV trust is adjusted.

(2) $$Trust\left(i,j\right)=\left\{\begin{array}{cc} \left[\left(1-\frac{1}{\#int\;+\;1}\right)\cdot DT\left(i,j\right)\right]+\left[\frac{1}{\#int\;+\;1}\cdot IT\left(i,j\right)\right] & if\;No\_GS\\ GSE(GS,j) & Else \end{array}\right.$$

DT(i,j) and IT(i,j) are the direct and indirect trust evaluation, respectively, calculated by a UAV *i* concerning another UAV *j*. The computation details of DT(i,j) and IT(i,j) are provided in the following sections.

#### 3.1.2. Direct Trust

The direct trust evaluation in our work is computed using the number of legal (*L*) and malicious (*M*) interactions between two UAVs *i* and *j* and it is computed following Equation (3):

(3) $$DT\left(i,j\right)=\frac{\#L(i,j)}{\#M(i,j)+\#L(i,j)}\cdot \left[1-\frac{1}{L(i,j)+1}\right]$$

where L(i,j) and M(i,j) represent the number of legal and malicious actions, respectively, between *i* and *j* from the perspective of the UAV *i*. $\frac{\#L(i,j)}{\#M(i,j)\;+\;\#L(i,j)}$ represents the percentage of legal actions compared to the total number of actions, and $1-\frac{1}{L(i,j)\;+\;1}$ is a factor approaching 1 when the number of legal actions increases. Hence, many legal actions are required to increase the direct trust to respect the general rule that says trust must be hard to win and easy to lose.

An action is considered legal or malicious based on a similarity measurement between the effective action taken by a UAV *j* and the same action simulated from the point of view of the UAV *i*. Based on the semantic kernels method [29], we use the function ϕ(x) to represent an action *T* having *n* features.

$$\phi :d\mapsto (t_{1},t_{2},\cdots ,t_{n})$$

$t_{1}$ is one of the features (1≤t≥n) and *n* is the total number of features of the action *t*. Similarity of two action T1 and T2, $\hat{k}\left(T1,T2\right)$ is given by Equation (4).

(4) $$\hat{k}\left(T1,T2\right)=\langle \frac{\phi (T1)}{∥\phi (T1)∥},\frac{\phi (T2)}{∥\phi (T2)∥}\rangle =\frac{k(T1,T2)}{\sqrt{k(T1,T1)k(T2,T2)}}$$

To compute k(T1,T2) we use the following formula:

$$\begin{array}{cc} k(T1,T2)=\langle \phi (T1),\phi (T2)\rangle & =\phi_{u}\left(T1\right).\phi_{v}\left(T2\right)\\ =\left(\sum_{T1:u=\phi (T1)}1\right)\left(\sum_{T2:v=\phi (T2)}1\right)\\ =\sum_{(T1,T2):u=v}1 \end{array}$$

where *u* is a feature of T1 and *v* is the same feature for T2. Finally, if the similarity is higher than 0.5 the action made by *j* will be considered legal, otherwise malicious.

#### 3.1.3. Indirect (Recommendation-Based) Trust

**Figure 4:**
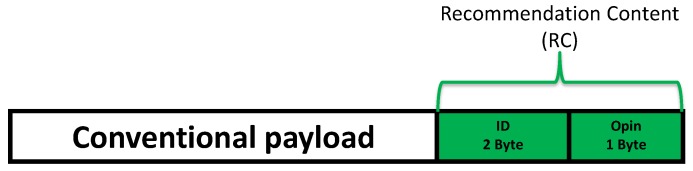
Piggybacking of Recommendation Contents.

Indirect trust is calculated based on recommendations coming from trusted UAVs about other unknown/known UAVs. Most of the existing solutions suggest creating a new message type called *recommendation*, and they choose either a cluster-based technique or an aggregation method to reduce the additional overhead. To avoid affecting the communications bandwidth and UAVs energy, we propose modifying the format of the exchanged data messages by adding only two fields: (i) the neighbor identity and (ii) the opinion of the sender about that neighbor. For example: if a UAV *i* considers that a UAV *j* is untrusted, it will put the UAV *j*’s identity within the data message along with an opinion which can be <0.5 (untrusted node) or >0.5 (trusted node). This opinion correspond to the global trust evaluation of the recommender about the recommended node Trust(i,j). Figure 4 illustrates the new messages format.

To avoid the negative influence of dishonest UAVs’ opinions on the indirect trust, a UAV *i* computes the trade-off between the trust and opinions of different recommenders. Hence, the higher is the level of trust of a neighbor, the more is its opinion taken into account. Equation (5) shows how the indirect trust is computed by a UAV *i* about another UAV *j*.

(5) $$\begin{array}{c} \forall k\in \{trusted\;direct\;neighbors\;of\;i\}\\ IT\left(i,j\right)={\left[\prod_{N}{\left(DT(i,k)\cdot Opin(k,j)\right)}^{\frac{1}{2}}\right]}^{\frac{1}{N}} \end{array}$$

In this equation, *N* refers to the number of recommenders, IT(i,j) is a combination of the recommenders’ (*k*) direct trust DT and their opinions about the UAV *j*. In addition, we consider a neighbor UAV as a trusted UAV if its trust Trust(i,j) is higher than a predefined threshold which can be adapted to the security requirements and the traffic type.

### 3.2. GroundStation-to-UAV Trust

On every interaction with the ground station, UAVs send their neighbor trust evaluation list to the ground station. This information allows the ground station to have a quasi-global view about UAVs behavior within their respective region. Equation (6) shows how a ground station computes its evaluation (GSE) of a UAV *j* using the other UAVs’ trust evaluation:

(6) $$GSE\left(GS,j\right)={\left[\prod_{n}Trust\left(i,j\right)\right]}^{\frac{1}{n}}$$

where *n* is the number of neighbors UAVs of *i* having a trust value regarding the UAV *j*.

In addition to the evaluation computed by GS, the received reports can also be used to detect and blacklist attackers within the network.

(7) $$\begin{array}{c} GSBlacklist\leftarrow \forall j,\\ \frac{Card(Trust\to ID\;=\;j\;\&\;Trust\to value\;\leq \;0.5)}{all\;reports}\geq DetectTH \end{array}$$

In Equation (7), DetectTH is the detection threshold, which is compared to the ratio between the number of UAVs considering the UAV *j* as dishonest and the total number of reporting UAVs.

### 3.3. Link Duration Estimation (LD)

Although the use of GPS should become commonplace in mobile nodes, we introduce a scheme to estimate the LD without the need of GPS (in case the GPS is not able to effectively estimate the velocity of nodes or is simply not available). We use the Doppler shift subjected to packets to calculate the relative velocity of nodes. The distance between nodes is calculated using the scheme used in [30], which uses signal strength to calculate the distance between the nodes by using the simplified free space propagation model given in [31]. For the mobility model it is assumed that mobile nodes are pseudo-linear, and highly mobile in nature which is the case for FANETs.

(8) $$LD\left(i,j\right)=\left\{\begin{array}{cc} \frac{1}{2v}(\sqrt{2d^{2}-4\left(d^{2}-R^{2}\right)}+d\sqrt{2} & \left(\frac{f}{f_{0}}<1\right)\\ \frac{1}{2v}(\sqrt{2d^{2}-4\left(d^{2}-R^{2}\right)}-d\sqrt{2} & \left(\frac{f}{f_{0}}>1\right) \end{array}\right.$$

where *f* is the actual frequency of the signal, $f_{0}$ is the observed frequency, *R* is the maximum communication range of the UAVs and *d* is the initial distance between two nodes given by:

(9) $$d=\frac{\lambda }{4\pi }\sqrt{\frac{P_{t}}{P_{r}}}$$

where $P_{r}$ is the initial received signal power, $P_{t}$ is the known transmission signal power and λ is the carrier’s wavelength. The link duration estimation for MANET can be also applied for FANET because of the continuous existence of Line of Sight (LoS) among communicating nodes [32].

### 3.4. FANET Distributed Monitor Selection and Monitoring Process

**Figure 5:**
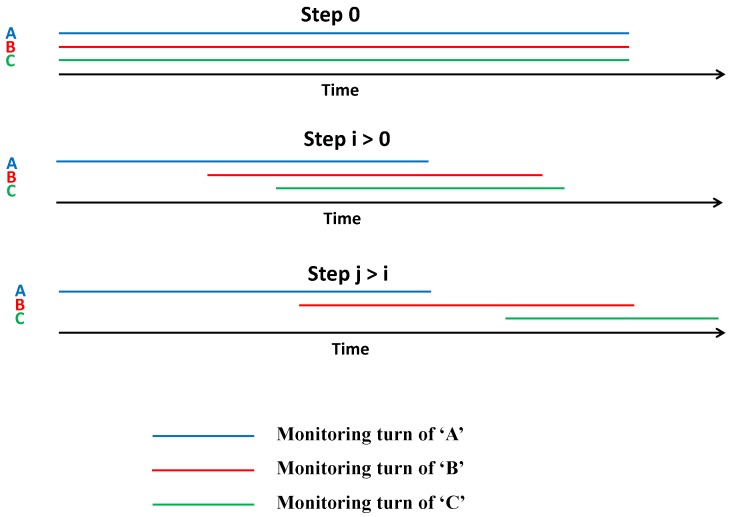
Convergence of our trust-based time division distributed monitoring technique.

In our proposal, network participants start by jointly monitoring the network from the beginning. Afterwards, the network monitoring task is distributed among peers trusting each other and hence, they can save their energy during the time other nodes are performing monitoring, as shown in Figure 5.

**Figure 6:**
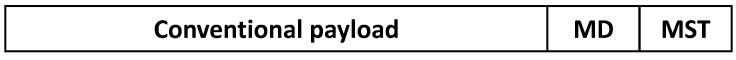
Hello messages format.

In order to make the monitoring process fully distributed among honest UAVs, we have extended the hello messages format with two new fields, MD and MST, respectively (see Figure 6).

After a certain number of interactions, the trust table of every node will be updated. Thus, every node will start trusting a certain number of neighbors. At this stage, different nodes can start distributing the monitoring process among them by sharing their MD along with the MST through the use of the extended hello messages.

Every node computes its monitoring duration using the number of trusted neighbors and the duration of the links towards them in such a way that the more trusted neighbors a node has, the lower becomes its monitoring time. Equation (10) outlines the monitoring duration computation.

(10) $$MD\left(i\right)=\left\{\begin{array}{cc} 0, & \;\mathrm{if}\;\#Neighbors=0\\ \frac{AVG[R\left(i,k_{1}\right),\cdots ,R\left(i,k_{n}\right)]}{\left[\frac{\#TrustedNeighbors}{\#Neighbors}\;\times \;\frac{1}{\#TrustedNeighbors+1}\right]}, & \;\mathrm{else} \end{array}\right.$$

In this equation, we multiply the average link duration by factor ${\left[\frac{\#TrustedNeighbors}{\#Neighbors}\times \frac{1}{\#TrustedNeighbors+1}\right]}^{-1}$, which represents the ratio of the trusted direct neighbors. Hence, the higher is this ratio, the lower is the monitoring duration. Otherwise, when a UAV is isolated, it does not have to stay in promiscuous mode and evaluate an empty neighborhood. Hence, the monitoring duration is set to 0. In addition, when a UAV receives different MD and MST values from its neighbors, it computes its next monitoring starting time using the last starting time and the monitoring duration of its neighbors as follows:

(11) NextMST(i)=LastTrustedNeighbors(MST+MD)

Once a monitor detects a malicious node, which means that the malicious node trust value goes below a detection threshold, it broadcasts a one-hop negative recommendation that includes the detected node identity. The receivers of this recommendation will autonomously adjust the indirect trust as previously explained.

## 4. Proposed Trust-Based Lightweight Authentication Strategy

In vanilla NDN, trusted self-signed authorities are always reachable. Hence, a decision about a data packet authenticity can always be made. However, such energy and time consuming strategy may not be feasible in FANETs for several-fold reasons such as mobility, network fragmentation, and battery restriction, to name a few. Furthermore, this strategy adversely affects the functioning of real-time and delay sensitive FANET applications such as disaster management. To address these problems, we propose a mechanism where we use the established trust among nodes to check the data authenticity. The established trust helps in deciding whether to bypass the authenticity check and accept the data without check.

For the data coming from an untrusted UAV, the traditional vanilla NDN authentication process is maintained in FNDN in order to ensure maximum confidence in the received data. However, for trusted UAVs, content authenticity is periodically checked offline, and the higher trust of the producer, the longer is the period of forwarding without check. Finally, for data coming from the ground stations, the normal NDN data authentication is automatically used. Therefore, checking the data authenticity of the already authenticated data through the wired part of the link, is not needed.

**Figure 7:**
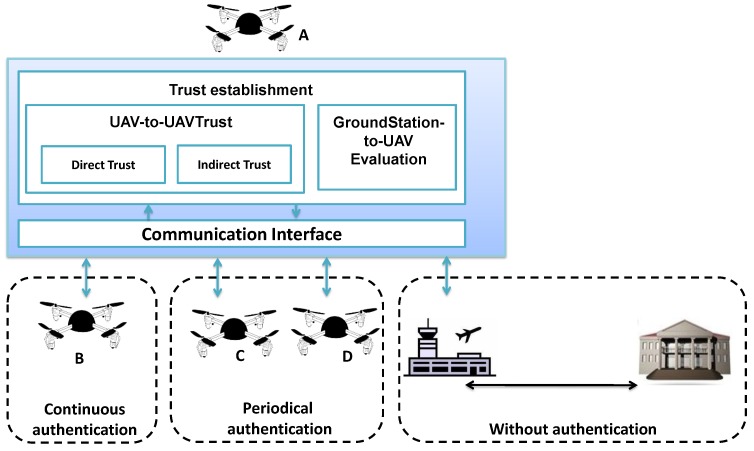
Overview of the proposed Trust-based data authentication mechanism.

**Figure 8:**
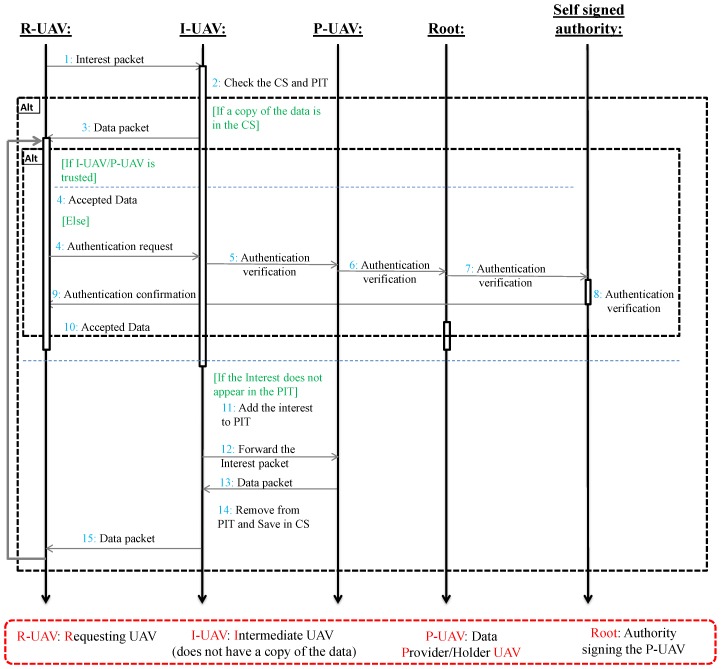
Sequence diagram of the proposed lightweight data authentication mechanism.

Figure 7 gives an overview of our proposed adaptive authentication process. In the figure, UAV *B* has a lower trust value (not trusted), therefore data received from *B* must be authenticated every time it is received by node *A*. Finally, the sequence diagram showing a step-by-step inter-UAV communication is presented is Figure 8.

## 5. Performance Evaluation

To evaluate performance of our proposed scheme, we used the NDN daemon implemented in the NS-3 simulator (ndnSIM) considering the IEEE 802.11p standard. The theoretical communication range of UAVs is assumed to be 400 m radius of the sphere created around their positions. As for the mobility model, UAVs are moving following the 3D random Waypoint Mobility Model with roughly 30 m altitude. In addition, since the current NDN implementation is over the routing layer, obtained results are not affected by the used mobility model. The topology region is considered to be 5 km${}^{2}$ in the simulations. Furthermore, the results are average of 10 simulation runs, where in each run 10 requesting UAVs or consumers generate interests at the rate of 20 interests per second. In addition to that, 4 data producers and 10% malicious UAVs are considered in each scenario. Those malicious UAVs inject unauthenticated data when they receive the interest packet. The consumer and producer UAVs are randomly chosen in every run. The total simulation duration is 600 s and the consumers generate interests during the whole simulation duration.

### 5.1. Performance Metrics

The performance evaluation is done through two security-related metrics and two network-related metrics, which are described below:

- *Detection ratio (%)*: It represents the ratio of the number of detected unauthenticated data packets to the total number of received unauthenticated data.
- *False positive (FP) ratio (%)*: The FP represents the number of data packets that are detected as unauthenticated while these packets are benign, to the total number of detected unauthenticated data.
- *Average end-to-end delay (s)*: It represents average time between sending the interest packet and receiving content as a result of the request for all connections.
- *Content delivery ratio (CDR) (%)*: CDR means the number of successfully fulfilled interest packets to the total number of generated interests.
- *Energy efficiency (W.s)*: It shows the average consumed energy for both strategies with and without the distributed monitoring.
- *Generated Overhead (Kb/s)*: It represents the additional trust establishment fields.

**Figure 9:**
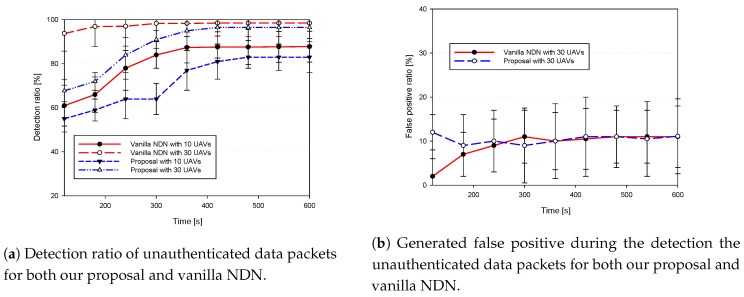
Security-related performance of both NDN and our proposal.

**Figure 10:**
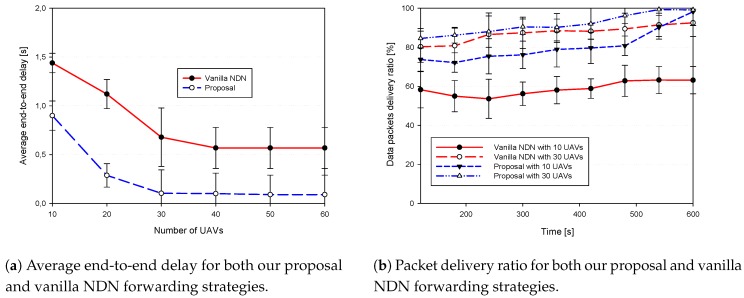
Network-related performance of both Vanilla NDN and our proposal.

**Figure 11:**
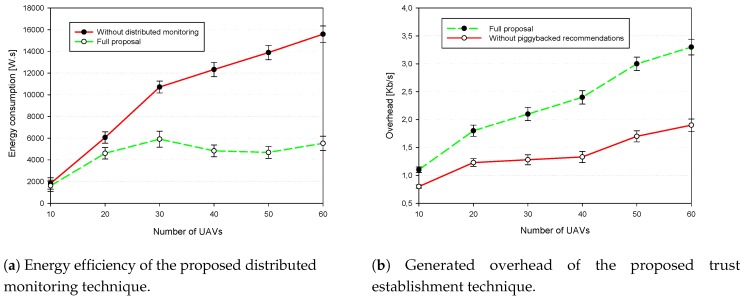
Generated overhead and energy efficiency our proposal.

First, we discuss the proposed trust-based data authentication strategy as compared to the traditional strategy implemented by vanilla NDN together with their generated false positive as shown in Figure 9. Afterwards, we study their ability to cope with delay-sensitive applications where the average end-to-end delay and the CDR as shown in Figure 10. Finally, we show through Figure 11, the average energy consumption and the generated overhead.

### 5.2. Results and Discussion

Figure 9a represents the ratio of the detected unauthenticated data packets for both, our proposed scheme and NDN authentication strategies in respect to the time for respectively 10 and 30 UAVs. Resulted curves show that for low density both strategies show less detection efficiency as compared to the high density, i.e., always in favor of the vanilla NDN strategy. However, after a short period of time (required to establish trust among UAVs); our proposal proposed scheme shows almost the same results as NDN strategy.

Additionally, Figure 9b represents the generated false positive and it shows that, except for the beginning of the experiments where inter-UAVs trust did not reach yet its stable values, the generated false positive is almost the same for both NDN and our proposal. From both the figures, it is clear that the trust-based data authentication strategy of our proposed scheme sustained almost the same performance of the vanilla NDN strategy with less delay and energy consumption for our proposal thanks to the distributed monitoring technique and the trust-based data authentication.

On the other hand, Figure 10a presents the average end-to-end delay of data packets and it shows that our proposed scheme reduces the delay by more than 300% as compared to vanilla NDN for 30 UAVs density. Whereas, this enhancement is around 80% for low density cases (i.e, 10 UAVs). Thus, the results advocate that our proposal outperforms the vanilla NDN and makes our proposal more suitable for real-time applications as well as the different FANET applications.

Figure 10b shows the packet delivery ratio for both our proposal and vanilla NDN forwarding strategies. It depicts that for both low and high density scenarios, our proposal trust-aware forwarding strategy outperforms vanilla NDN forwarding strategies, showing almost optimal results for high density case.

Figure 11a analyzes the energy consumption of our proposal. We analyzed the scenario with 20% of malicious UAVs also for both cases with and without the distributed monitoring process in respect of the network density. Resulted curves show that while sustaining the detection performances, our distributed monitoring technique have reduced the average energy consumption by more than two times for high density cases, for instance in the case of 50 UAVs the average consumed energy is about 14,000 W.s for the continuous promiscuous mode. Whereas using our solution the average consumed energy becomes about 4400 W.s which is a significant optimization.

Finally, for the introduced communication overhead with the piggybacked recommendations, Figure 11b depicts that our strategy of piggybacking the trust opinion to the data messages introduces increased values with the increasing number of UAVs and this is due to the consideration of all trusted nodes opinions to strengthen the detection process, and theses trusted UAVs are majority in the normal case.

## 6. Challenges in FNDN Security

Vanilla NDN brought various security challenges to its architecture like interest flooding, cache poisoning, key management problems. As we mentioned in the introduction that data/content must be signed by the producer and then hierarchically by the authorities that signed the certificate of the producer, it implicitly ensures three basic security features that are: data integrity, correctness, and its source authentication and thus, provides data centric security rather than securing communication channel. Here we discuss several security issues in FNDN that need further investigation.

### 6.1. Efficient Key and Identity Management

Existing works discussed only how efficient curretly used signature algorithms (such as SHA256 with RSA, ECDSA, or HMAC) are? However, very few works addressed the problem of certificate distribution, revocation techniques, and pseudonyms changing to preserve producers and their data privacy. Moreover, in FNDN the conventionally trusted authorities may not be reachable sometimes. So, how will the data authenticity be checked? More in depth investigation is needed to check the feasibility of currently available schemes in FNDN.

### 6.2. Network Performance

The current Vanilla NDN works over IP and the NDN packets are already embedded into the lower layers that have their own security mechanisms. The effect of this double check for authenticated data would also decrease the network performance. In order to address this issue, more information is needed for lower layer to bypass the data authenticity when needed. Hence the security schemes for the current NDN are subject to further research.

### 6.3. Data Security

Data packet is signed but not encrypted which means that data confidentiality is not guaranteed. For now, we suppose that the requested data is freely available; however if the data is encrypted, then key management and access to that data should be provided to the consumer based on the access rights. Another problem is the content store where the content could be cached in the encrypted form, but the meta information about the content would still be in a cleartext. Therefore, more investigation is needed to handle access to the encrypted data in NDN. This issue is more serious in FNDN due to the mobility and intermittency among UAVs.

### 6.4. Access Control

Access control is one of the most important requirements in FNDN. When consumers send interest for data, it should be checked whether the consumer has access rights for that content or not. Another important aspect is the entity who is responsible to validate the access rights, whether it should be the publisher or any intermediate node? In wired networks, usually intermediate routers are responsible for such verifications; however, in mobile networks such as FNDN, this security aspect needs in-depth investigation.

### 6.5. Trust Management

Different from traditional TCP/IP-based trust management for FANET where trust is divided into entity-based, data-based, and hybrid trust model, FNDN by nature, only supports data-based trust model. Thus, efficient data trust management schemes are essential in FNDN. It is worth noting that entity trust cannot be completely ignored in FNDN because the data trust must be linked to the publishers/producers; however, due to mobility and intermittent connections, managing trust level for individual nodes will incur huge overhead. Therefore, an efficient alternative must be developed. Furthermore, trust lifetime is another aspect that needs further investigation. Depending on the type of application, context-aware trust management scheme could perform better in FNDN where the trust parameters could be changed based on the context of the application. To this end, both delay-sensitive and delay-tolerant applications must be taken into account while working with trust.

### 6.6. Trust Bootstrapping and Propagation

Another trust related issue is the bootstrapping. Usually, an average value is assigned to the nodes on their first encounter but that does not necessarily mimic the actual behavior of the node. Therefore, historical information of the nodes should be shared among the neighbors to evaluate the trust. Furthermore, trust propagation will be challenging in FNDN because it may increase the network overhead. However, piggybacking approach can be investigated to see its effectiveness in trust propagation in FNDN.

### 6.7. Auditing and Incentives

Trust propagation and recommendation require cooperation among neighboring nodes. In resource-constrained and intermittent environments, this propagation becomes challenging and it will be difficult to convince neighbors for recommendation. Therefore, in order to stimulate the active participation of the neighbors for recommendation, incentives could be introduced as in the traditional networks; however, the communication paradigm of NDN advocate for new mechanisms to manage incentives and the audit of the incentives. More research is needed in this direction. To sum up, FNDN is a promising communication paradigm to realize ITS applications and services; however, security and privacy issues must be extensively investigated and addressed before its commercialization.

## 7. Conclusions and Future works

Despite the success of Vanilla NDN, security and privacy issues still need to be addressed. The intrinsic principle of security in NDN and its breeds such as VNDN have caused other issues such as the overhead incurred by hierarchical data authenticity. In principle, hierarchical data authenticity eliminates all types of man-in-the-middle attacks but at the expense of huge computational, storage, and delay overhead, mostly undesired in highly mobile and energy-restricted networks such as FANET. To fill the voids, we proposed a novel Trust-Aware Monitor-based Communication Architecture for Flying Named Data Networking. Our proposed scheme leverages a trust-aware Network Monitoring technique to avoid the broadcast storm problem during the interests dissemination. Our proposal uses the inter-UAV trust to choose whether to check the data authenticity for a particular node or not without affecting the desired security levels. Simulation results proved the efficiency of our solution at sustaining Vanilla NDN security levels together with a clear overhead and energy minimization. The paper also discussed main open challenges in FNDN security.

As future work we plan to investigate mainly the data privacy and caching policies. In addition, we also plan to investigate the trustworthiness of the data provided by the heterogeneous vehicular networks involving different kinds of technologies and features.
